# Reflectivity and Angular Anisotropy of Liquid Crystal Microcapsules with Different Particle Sizes by Complex Coalescence

**DOI:** 10.3390/molecules29133030

**Published:** 2024-06-26

**Authors:** Yonggang Yang, Yuchen Cui, Yinjie Chen, Yanan Guo, Xiaoqi Liu, Xia Chen, Jianghao Liu, Yu Liu, Zhengfeng Liu

**Affiliations:** 1 School of Printing and Packaging Engineering, Beijing Institute of Graphic Communication, Beijing 102600, China; yangyonggang@bigc.edu.cn (Y.Y.); 18339652952@163.com (Y.C.); 18811515067@163.com (Y.G.); lxqdyx517@163.com (X.L.); arisliu@bigc.edu.cn (J.L.); liuyu@bigc.edu.cn (Y.L.); liuzhengfeng@bigc.edu.cn (Z.L.); 2 Beijing Engineering Research Center of Printed Electronics Institution, Beijing Institute of Graphic Communication, Beijing 102600, China; 3 School of New Media, Beijing Institute of Graphic Communication, Beijing 102600, China; chenxia@bigc.edu.cn

**Keywords:** cholesteric liquid crystal microcapsules, complex coacervation, reflectivity, angular anisotropy

## Abstract

Cholesteric liquid crystal microcapsules (CLCMs) are used to improve the stability of liquid crystals while ensuring their stimulus response performance and versatility, with representative applications such as sensing, anticounterfeiting, and smart fabrics. However, the reflectivity and angular anisotropy decrease because of the anchoring effect of the polymer shell matrix, and the influence of particle size on this has not been thoroughly studied. In this study, the effect of synthesis technology on microcapsule particle size was investigated using a complex coalescence method, and the effect of particle size on the reflectivity and angular anisotropy of CLCMs was investigated in detail. A particle size of approximately 66 µm with polyvinyl alcohol (PVA, 1:1) exhibited a relative reflectivity of 16.6% and a bandwidth of 20 nm, as well as a narrow particle size distribution of 22 µm. The thermosetting of microcapsules coated with PVA was adjusted and systematically investigated by controlling the mass ratio. The optimized mass ratio of microcapsules (66 µm) to PVA was 2:1, increasing the relative reflectivity from 16.6% (1:1) to 32.0% (2:1) because of both the higher CLCM content and the matching between the birefringence of the gelatin–arabic shell system and PVA. Furthermore, color based on Bragg reflections was observed in the CLCM-coated ortho-axis and blue-shifted off-axis, and this change was correlated with the CLCM particle size. Such materials are promising for anticounterfeiting and color-based applications with bright colors and angular anisotropy in reflection.

## 1. Introduction

Cholesteric liquid crystals (CLCs) exhibit a structural color of selective reflection owing to the helical arrangement of the molecules [1]. However, their inherent fluidity limits their ease of processing and structural stability, and numerous methods, such as cholesteric liquid crystal microcapsule (CLCM) technology, have been adopted to achieve their functionality [2,3]. In contrast to planar CLC films, CLCMs consisting of an inner liquid crystal as the core material and an outer polymer as the shell material, which turn the liquid crystal system into a quasi-solid state, have attracted notable interest because of their great advantages in various applications [4,5,6,7,8,9,10]. In this regard, CLCMs present a low-viewing-angle-dependent color shift in a practical sense because they are physically constrained by their spherical shell. When the capsule is somewhat small, it exhibits angular isotropy. Moreover, CLCMs avoid the large-area crystallization of hybrid liquid crystals that can occur at the operating temperatures of nonencapsulated liquid crystals, thus greatly expanding their range of use [11]. In addition, the microencapsulated structure of CLCs is beneficial for ink processing. However, the presence of an external shell material has an adverse effect on the optical transmittance, the stimulus response to electric fields, light, magnetism, and temperature, and the mechanical properties of liquid crystal microcapsules, restricting their further application.

Various methods and polymer shell materials are currently used to synthesize microcapsules [12,13,14,15,16,17,18,19,20,21,22,23,24,25]. The first reported microcapsule technology to coat liquid crystals, the complex coalescence method, is now mature and is still one of the most effective and widely used ways to prepare liquid crystal microcapsules owing to the natural properties of the gelatin–arabic (G-A) system, which is nontoxic and biocompatible [26]. Pioneering studies have been conducted on a complex condensation method to prepare liquid crystal microcapsules for photo-responsive [21], drug release [25], and color rendering performance research [27]. Guan et al. (2018) [23] prepared temperature-responsive liquid crystal microcapsules with 14% reflectivity using the complex coalescence method and used them in thermochromic fibers. Yang et al. (2022) [28] increased the reflectivity of CLCMs to 35% by adding a cellulose nanocrystal orientation layer to help orient liquid crystal molecules. It has been reported that CLCMs prepared by the microfluidic method usually have higher reflectivity than those prepared by other methods because of the ultraviolet monomer acting as the microcapsule shell [29]. For example, Lin et al. (2019) [30] used microfluidics to prepare blue-phase liquid crystal microcapsules and constructed a color library with a wide color gamut and a reflectivity of up to approximately 40%. However, the high manufacturing and time costs of microfluidics limit its large-scale applications. In addition, the choice of the shell layer material significantly affects the anchoring effect of the interface, and the anchoring effect of the shell varies with shell thickness, which interferes with the reflectivity of the liquid crystals [12,31]. Few researchers have analyzed the effect of particle size on the reflectivity and angular anisotropy of CLCMs in detail.

In this study, CLCMs with different particle sizes were prepared by complex coalescence. The influence of reflectivity was investigated in detail based on the synthesis conditions and coating ratio, resulting in highly saturated CLCMs with 32% relative reflectivity and 20 nm half-wave widths, as well as a narrow particle size distribution. The coating was prepared by mixing transparent and colorless polyvinyl alcohol (PVA) with microcapsules. The optimal ratio of microcapsules to PVA was 2:1, owing to both the higher CLCM content and the match between the birefringence of the G-A shell system and PVA. Coatings of different sizes were prepared by scratch coating. The wavelengths of CLCM coatings with particle sizes in the range of 34–80 µm showed a blue-shifted reflection at viewing angles that were off the positive axis of the coating. The results provide insights into the development of stimulus-responsive nanostructures and new applications in diverse fields ranging from sensing to displays.

## 2. Results and Discussions

**Figure 1:**
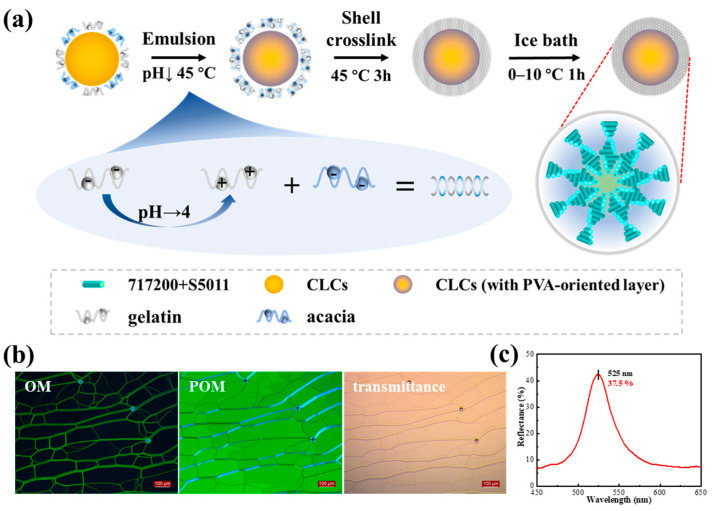
(**a**) Schematic of the fabrication procedure of CLCMs by complex coacervation; (**b**) OM, POM, and transmission images of CLCs in parallel oriented cell and its reflection spectrum (**c**).

Figure 1a shows a schematic of CLCM fabrication. The nematic liquid crystal 717200 and chiral agent S5011 were chosen as core materials. The liquid crystal 717200, with a small birefractive index (∆n=0.039), was selected as the main component of the CLCM to obtain a narrow half-wave width and highly saturated samples. S5011 was selected as the chiral agent because it is insensitive to temperature and has a high spiral twisting force constant ($HTP=104\;{\mu m}^{-1}$). The compositions of the CLCMs used are listed in Table 1. Among them, CLCM A2, B3, C3 and G were the products of one experiment. The configured liquid crystals showed a characteristic oily streak texture for the cholesteric phase in the cell and bright color based on Bragg reflections (Figure 1b), and the chiral agent was used to control the reflection wavelength, which had a strong reflection peak of approximately 37% at 525 nm in the cell (Figure 1c). The chemical structures of chiral compound S5011 can be seen in Figure S1.

A complex coacervation was used to fabricate the microcapsules. Arabic and PVA 1788 were used together as emulsifiers to disperse the core materials. The liquid crystal was dispersed into small droplets at a high speed of 20 min at 45 °C. After the pH is adjusted, because acacia molecules are negatively charged at the oil–water interface, the positively charged gelatin molecules will be evenly distributed on the surface of the oil drop due to electrostatic action, resulting in the water phase being wrapped in the oil phase. Gelatin contains carboxylic $\left(COOH⇌{COO}^{-}+H^{+}\right)$ and amine $\left({{NH}_{3}}^{+}⇌{NH}_{2}+H^{+}\right)$ groups as ionizable groups and is therefore highly pH-dependent in the reaction. After 3 h of reaction, the microcapsule had a preliminary putaminal structure. In the final step, aldehydes were used to solidify the shells of the microcapsules at less than 10 °C, where the aldehyde group of the aldehydes underwent a condensation reaction with the amino group in the gelatin molecule to enhance the surface strength of the microcapsules. The characterization and analysis results are shown in Figure S2.

### 2.1. Effect of Spin Speed and Rotor Size

**Figure 2:**
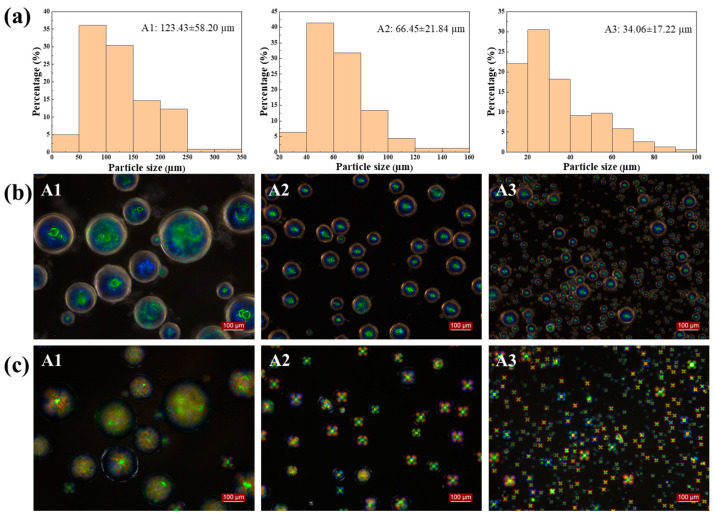
(**a**) Particle size distribution of CLCM A1-A3; (**b**) OM and (**c**) POM images of CLCM A1–A3.

Spin speed plays an important role in controlling the particle size and distribution of CLCMs. Figure 2 shows that as the spin speed increased from 500 to 1000 to 1500 rpm, the average diameters of CLCMs decreased from 123.43 to 66.45 and 34.06 µm, respectively, the standard deviation decreased from 58.20 to 21.84 to 17.22 µm, and the coefficient of variation changed from 47.2 to 32.9 to 50.6%, respectively. This is because as the spin speed increased, the shear force in the system became larger, which caused the droplet cores to be dispersed into smaller droplets. When the average particle size of the CLCMs was 123.43 µm, the “Maltese” phenomenon was hardly observed by polarized optical microscopy (POM); however, when the particle size decreased to 34 and 66 µm, the image showed a bright Maltese pattern (Figure 2c). This is because a large microcapsule is detrimental to the radial arrangement of the liquid crystal molecules, presenting an internal chaotic molecular orientation owing to the weakened binding force of the shell. When the spin speed was fixed at 1000 rpm, the microcapsules showed a bright, clear color, with an average diameter of 66.45 µm and a low coefficient of variation of 32.9%. Detailed particle size analysis and calculations are in Table S1.

**Figure 3:**
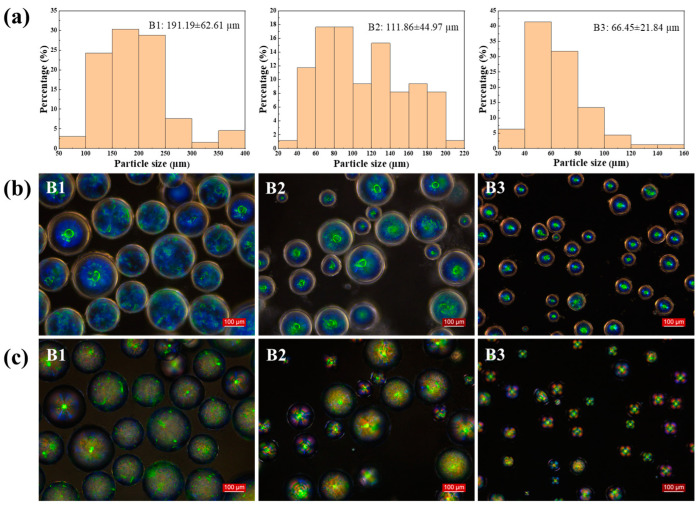
(**a**) Particle size distribution of CLCM B1–B3; (**b**) OM and (**c**) POM images of CLCM B1–B3.

To further elucidate the influence of this system, the spin speed was fixed at 1000 rpm, and the effect of the rotor size was investigated in detail. The rotor size had a more significant effect on the particle size of the microcapsules than the spin speed. Rotors with lengths of 2.0, 2.5, and 3.0 cm produced microcapsules with average particle sizes of 191.19, 111.86, and 66.45 µm, respectively (Figure 3a). The rotational strength of the small rotor was insufficient, resulting in a lower shear rate. This, in turn, affected the larger particle size of the microcapsules.

**Figure 4:**
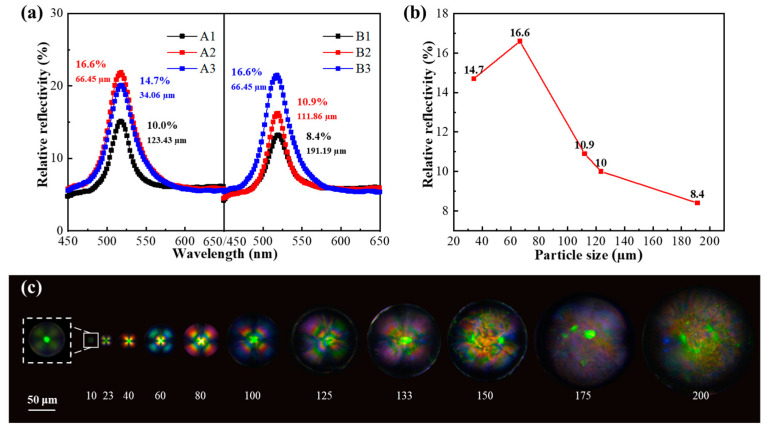
(**a**)The relative reflection spectra of CLCM A1–A3\B1–B3. (**b**) Comparison of the relative reflectivity of CLCMs with different particle sizes. (**c**) The POM images of CLCMs.

Figure 4a shows the dependence of the average particle size of the microcapsules on the reflection spectra. A 1:1 microcapsule to PVA ratio was selected for preparing the coating solution. The relative reflectivity of the CLCM coating increased (8.4%, 10.0%, 10.9%, 16.6%, and 14.7%) in inverse proportion to the average particle size (191.19, 123.43, 111.86, 66.45, and 34.06 µm), reaching the maximum value of 16.6% when the particle size was 66.45 µm. As the POM images in Figure 4c show, when the diameter of the microcapsule exceeded 100 µm, the specific surface area decreased, only the molecules on the surface of the inner shell were oriented, and the internal liquid crystal molecules of the CLCMs had an irregular arrangement, resulting in increased scattering and decreased reflectivity. This is consistent with the “Maltese” phenomenon in POM. A medium CLCM particle size of 40–100 µm is advantageous for displaying bright color. For microcapsules with smaller particle size, such as 34.06 µm, due to the smaller microcapsule size, the cholesteric liquid crystal spiral structure that can be accommodated inside becomes less; although high orientation can be obtained, it also affects the color rendering efficiency. This point can also be reflected in the POM image, although the “Maltese” phenomenon is very obvious, but the color is not bright. Therefore, a 3 cm rotor size and 1000 rpm spin speed were selected, resulting in a typical particle size of approximately 66 µm.

### 2.2. Effect of Core-to-Shell Ratio

**Figure 5:**
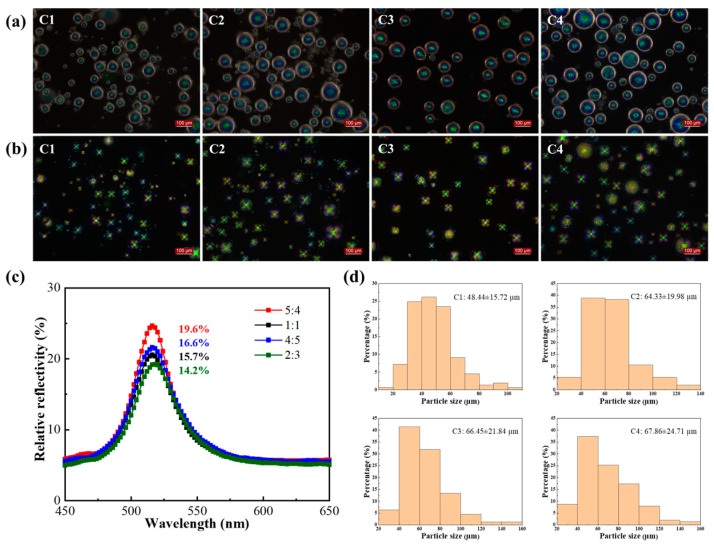
(**a**) OM and (**b**) POM images of CLCM C1–C4. (**c**) The relative reflection spectra of CLCM C1–C4. (**d**) Particle size distribution of CLCM C1–C4.

The core-to-wall ratio mainly affects the shell thickness and mechanical properties of CLCMs. As shown in Figure 5, when the core-to-shell ratios were 5:4, 1:1, 4:5, and 2:3, the average particle sizes of the CLCMs were 48.44, 64.33, 66.45, and 67.86 µm, respectively. When the shell material was less than the core material, that is, CLCM C1 (5:4), the particle size was significantly smaller than that of CLCM C2/3/4. Meanwhile, CLCM C1 appeared to rupture easily because less shell material resulted in a thinner shell. Although a larger proportion of shell materials results in higher mechanical strength, the relative reflectivity of Group C with PVA at a 1:1 ratio decreased from 19.6%, 15.7%, 16.6%, to 14.2%, respectively, owing to the interference of the gradually thickening shell material (Figure 5c). Comprehensively considering the mechanical properties and reflectivity revealed that CLCMs with a core-to-wall ratio of 4:5 are preferable owing to their high relative reflectivity of 16.6%.

In the above experiments, the particle sizes of the microcapsules depended mainly on the spin speed and rotor size. A higher core-to-shell ratio of the microcapsules led to smaller capsules with thinner shells, resulting in high reflectivity and low mechanical strength. Therefore, the optimal conditions for synthesizing microcapsules are as follows: spin speed of 1000 rpm, rotor size of 3.0 cm, and core-to-wall ratio of 4:5.

### 2.3. Effect of Emulsifiers and Curing Agent

**Figure 6:**
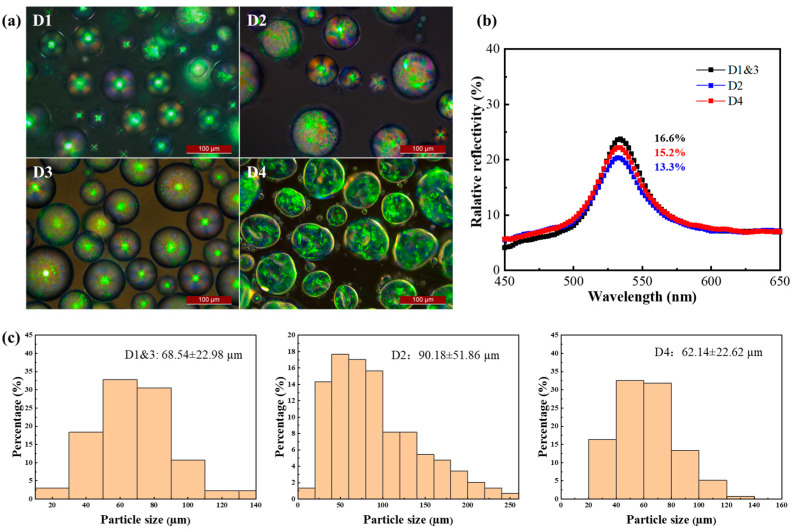
(**a**) The POM images of CLCM D1–D4. (**b**) The relative reflection spectra of CLCM D1–D4. (**c**) Particle size distribution of CLCM D1–D4.

The effects of emulsifiers and curing agents were investigated using POM, and the microcapsules with PVA as an emulsifier (CLCM D1), in contrast to those without PVA (CLCM D2), showed an ideal “Maltese” phenomenon under POM, which indicates a regular radial arrangement of liquid crystal molecules (Figure 6a). The coefficient of particle size variation of CLCM with PVA addition (33.5%) was lower than that of CLCM without PVA addition (57.5%), and the particle size was smaller (Figure 6b). The participation of PVA represents the increase in orientation order, and correspondingly, the relative reflectivity of CLCM also increases.

In addition, unlike formaldehyde (CLCM D4), glutaraldehyde (CLCM D3) does not require an alkaline-environment-induced reaction and can be cured under neutral conditions. Thus, it avoids the intense curing reaction that damages the microcapsule shell, which changes the protein molecules from the original ordered and compact structure to a disordered and lax structure. The effect of formaldehyde on particle size and reflectivity is lower than that of glutaraldehyde. The deformation of the microcapsule shell causes the internal arrangement order to be destroyed, which leads to the decrease in color rendering. The change in particle size is due to the shrinkage of the shell to a certain extent due to the intense polycondensation reaction (Figure 6). Detailed particle size data are shown in Table S2. Process conditions of CLCM D1–D4 are shown in Table 2.

### 2.4. Optical Property

**Figure 7:**
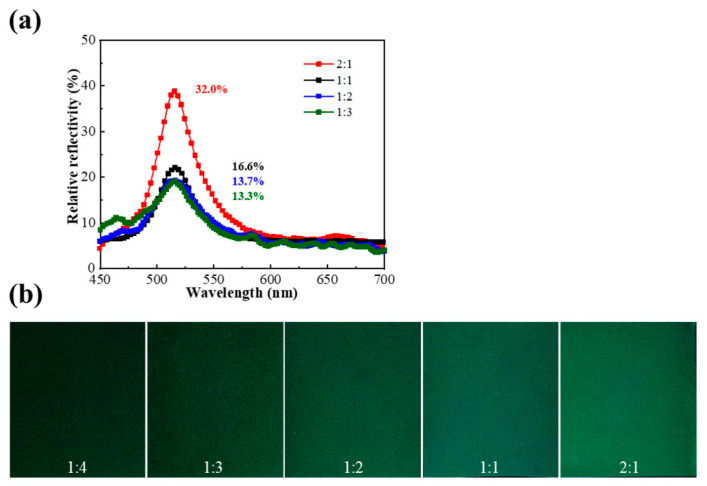
(**a**) The relative reflection spectra of liquid crystal coatings with microcapsules mixed with PVA in different ratios. (**b**) The photographs of liquid crystal coatings with microcapsules mixed with PVA in different ratios.

To gain further insight into the reflectivity, the effect of the ratio of PVA aqueous solution and CLCM contents on the color of the liquid crystal coating was investigated. As shown in Figure 7, the reflectivity increases in proportion to the added CLCM content. When the ratio of microcapsules to PVA was 2:1, a higher relative reflectivity of nearly 32% was obtained compared with previous results (Table 3), indicating both the high transparency of the shell materials and the matching of refractive indices between PVA (1.3520) and gelatin–acacia shell materials (1.3357). Subsequently, when the ratio was reduced to 1:4, the coating could not cover the PET substrate, resulting in strong light scattering, so the spectrometer could not measure the reflection spectra (Figure 7b). When the ratio of microcapsules to PVA exceeded 2:1, the CLCM solution was unsuitable for coating methods because it lacked rheology.

**Figure 8:**
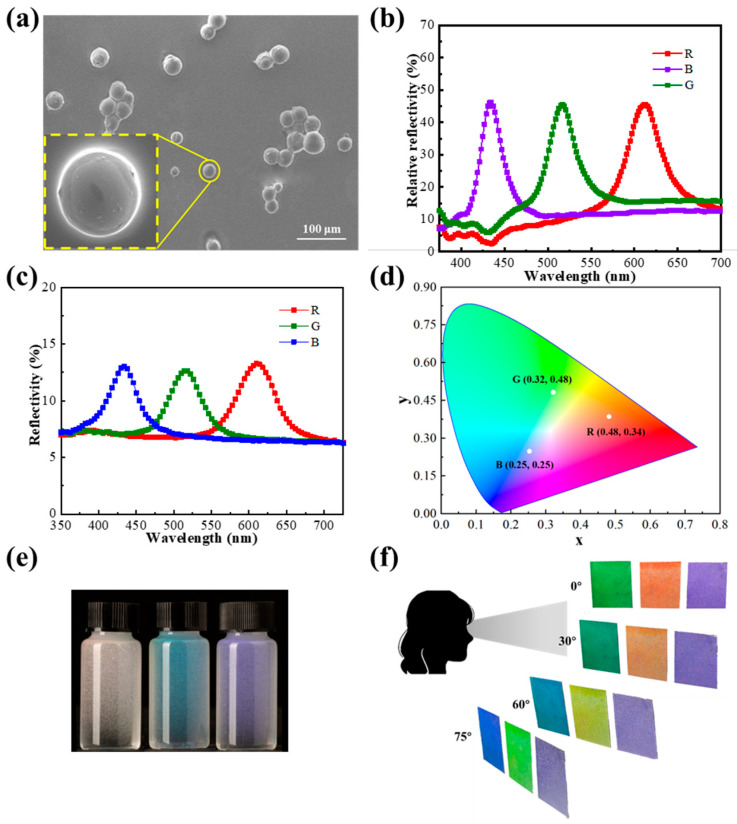
(**a**) SEM images of CLCM RGB. (**b**) The relative reflection spectra CLCM RGB. (**c**) The reflection spectra of CLCM RGB. (**d**) The chromaticity diagram of CLCM RGB. (**e**) The image of CLCMs in the aqueous solution. (**f**) The images of CLCM RGB coatings at different viewing angles.

Under the optimal experimental conditions, RGB trichromatic microcapsules were prepared, as shown in Figure 8a, where the spherical microspheres with full shapes and smooth surfaces were observed in scanning electron microscopy (SEM) images. OM and POM images are shown in Figure S3. The center wavelengths ($\lambda_{0}$) of RGB trichromatic microcapsules coated with PVA (2:1) were located at 612, 516, and 434 nm, accompanied by a high relative reflectivity of approximately 30% and a half-wave width of approximately 20 nm (Figure 8b). The absolute reflectivity was tested by a UV spectrophotometer (Figure 8c), and the coatings reached a value of 6% under optimal conditions. The center wavelengths of CLCs are inconsistent before and after encapsulation, and the center wavelengths of CLCMs (516 nm) are blue-shifted relative to CLCs (525 nm) in cells because of angular disturbances caused by the radial alignment of liquid crystal molecules in the globular structure, and $\lambda_{0}$ decreases according to Equation (1). The color coordinates of the CLCM RGB (Figure 8d) are located at (0.48, 0.34), (0.32, 0.48), and (0.25, 0.25), respectively. This suggests that the liquid crystal coatings have an appreciable color contrast, with chromaticity coordinates that are all relatively far from the central chromaticity coordinates (0.33, 0.33). Although the microcapsules showed the same color at different angles in the aqueous solution (Figure 8e), the coatings did not completely lose their angular anisotropy (Figure 8f). The prepared microencapsulated trichromatic coatings exhibited different colors at different viewing angles to the human eye. As shown in Figure 8f, when the line of sight was shifted away from the positive axis of the coating (i.e., the viewing angle was not 0°), the wavelength of the coating showed an overall blueshift. As the viewing angle is shifted from 0° to 75°, the red coating turns to orange, yellow, and light green; the green coating turns to dark green, lake blue, and royal purple; and the blue coating turns to dark purple, violet, and light purple. Among them, both red and green coatings showed significant color changes. The blue coating only shows a lightening of color due to the blueshift of the central wavelength to the ultraviolet region, which is beyond the range of color perception of the human eye.

**Figure 9:**
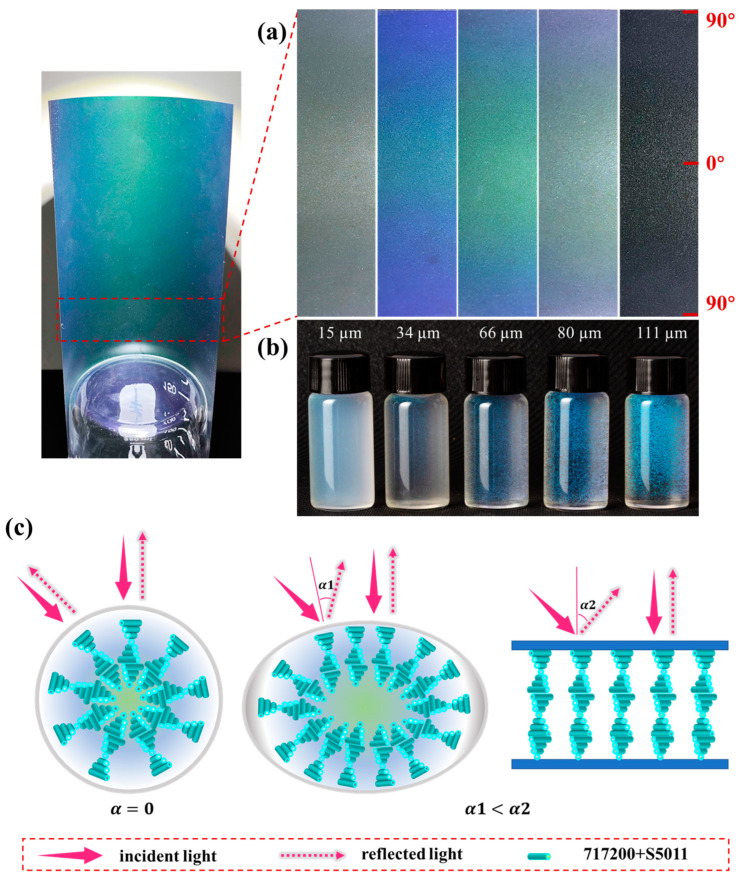
(**a**) Images of liquid crystal coatings at different viewing angles. (**b**) Images of microcapsules in aqueous solution. (**c**) The scheme of CLCMs in aqueous solution (**left**), CLCMs in coatings (**middle**), and CLCs in cell (**right**).

To elucidate the angular anisotropy and particle size of CLCM systems, microcapsule coatings of different particle sizes were investigated, as shown in Figure 9. The microcapsule coating with a particle size of 15 µm showed almost no color owing to the deficiency of internal cholesteric molecules. Furthermore, significant angular discoloration was observed for microencapsulated coatings with particle sizes of 34, 66, and 80 µm, with the coating being green when viewed straight on (middle of the image), whereas the coating turned out to be blue when viewed obliquely infinitely close to 90° (edge of image), as shown in Figure 9a. This could be related to the morphology of the microcapsules. After the aqueous coating was dried, the microcapsules squeezed downward and became flattened (Figure 9c), resulting in some liquid crystal molecules being aligned parallel inside the microcapsules, bring a certain angular anisotropy. According to Equation (1) [33],

(1) $$\lambda_{0}=\bar{n}P\cdot \cos\alpha$$

The CLCM color was not only related to its refractive index $\bar{n}$ and pitch P but also to the helical axis and the incident light angle α. Because the microcapsules have a limited degree of deformation in the coating, α in the cell is greater than it is in the coating (Figure 9c).

In addition, as the particle size continued to increase, the color and its angular anisotropy showed a downward trend. The angular anisotropy of microcapsules with a particle size of 111 µm is almost unobservable. On the one hand, microcapsules with too-large particle sizes are not suitable for coating. During the coating drying process, microcapsules will break, and the spilled microcapsules will be disorganized in the PVA, resulting in reduced color rendering efficiency. On the other hand, the material configuration of the coating is prepared by mixing PVA of the same quality with microcapsules. Moreover, the larger the particle, the fewer microcapsules per unit mass, which reduces the content of the liquid crystal, thereby sacrificing color.

The angular anisotropy of the coating is related to the degree to which the microcapsules are compressed. For the same coating thickness (140 µm), the average thickness of microcapsule coatings after drying with particle sizes of 15, 34, 66, 80, and 111 µm is 17.52, 13.85, 14.86, 15.06, and 19.55 µm (Figure S4). After drying, the average thickness of the coating was significantly reduced, even lower than the diameter of the microcapsules, indicating that the microcapsules had a large degree of compression during the drying process. In addition to the 15 µm sample, the trend in microcapsule coatings with other particle sizes is that the larger the particle size, the larger the average thickness of the coating. This is because the microcapsules with the smallest particle size of 15 µm have a large number of microcapsules per unit mass. A high proportion of shell mass is densely packed in the coating solution, so the thickness is higher after drying.

## 3. Experiment

### 3.1. Materials

The nematic liquid crystal (HNG-717200, T_N–I_ = 82.0 °C, Jiangsu Hecheng Display Technology Co. Ltd., Nanjing, China) and the chiral compound (S5011, Jiangsu Hecheng Display Technology Co. Ltd.) were used as the core materials. Gelatin and arabic were the components of the microcapsule shell materials, purchased from Shanghai Macklin Biochemical Co., Ltd. (Shanghai, China). PVA 1788 and glutaraldehyde were also purchased from Shanghai Macklin Biochemical Co., Ltd. All the chemicals were used as received without further purification. The olive-shaped PTFE stirrer was purchased from Hunan Beekman Biotechnology Co., Ltd. (Changsha, China).

### 3.2. Preparations of CLCMs

The gum arabic aqueous solution and PVA 1788 were added to a 250 mL round-bottom flask to form an aqueous phase, which was then placed in a 45 °C water bath. The 717200 and S5011 were dissolved in dichloromethane to form a homogeneous oil phase. Then, the oil phase was dropped into the aqueous phase to form a stable oil-in-water emulsion by the effect of emulsification through high-speed shear dispersion. An aqueous gelatin solution of the same quality as the gum was added after adjusting the pH to 4.0, which was then maintained for 3 h at 45 °C. An ice-water bath was used after natural cooling to room temperature, and then 1 mL of glutaraldehyde was added. The stirring rate was kept constant and maintained for 1 h during this polycondensation reaction of ammonia–aldehyde condensation. Finally, the microcapsules were rinsed with deionized water multiple times and treated with multiple centrifuges to remove excess impurities.

### 3.3. Preparation of Coatings

After centrifugation of the reaction solution, the resulting concentrated CLCM slurry was mixed with an aqueous PVA solution to prepare a coating solution, which was then applied to a black PET substrate using a squeegee coating machine (MSK-AFA-IIID, Hefei Kocrystal Material Technology Co., Ltd., Hefei, China) to form a liquid crystal coating.

### 3.4. Characterizations

The morphologies of microcapsules were observed by scanning electron microscopy (SU8020, Hitachi, Tokyo, Japan) and a polarizing optical microscope (DM2700M, Leica, Weitzlar, Germany). Particle size data were obtained by recording the diameter of a single microcapsule in the OM image, with at least 150 microcapsule diameters recorded for each sample. The composition of microcapsules was analyzed by Fourier transform infra-red spectroscopy (IS10, Nicolet, Green Bay, WI, USA). The relative reflection spectra and chromaticity value were measured using a fiber optic spectrometer (AvaSpec-2048, Avantes, Apeldoorn, The Netherlands). The absolute reflection spectra were measured using an ultraviolet spectrophotometer (UV-3600, Shimadzu, Tokyo, Japan). The reflectivity value was calculated by subtracting the baseline from the peak on the y axis. X-ray diffraction (XRD) data were obtained with a powder diffractometer (D/max-2200PC, Rigaku, Tokyo, Japan), using unfiltered Cu Kα radiation (*λ* = 1.5406 Å). The average coating thickness was measured by laser confocal microscopy (VK-X200K, Keyence, Osaka City, Japan).

## 4. Conclusions

In this study, CLCMs with core–shell structures were successfully prepared by complex coacervation. Under the optimal process conditions (a speed of 1000 rpm, a core-to-wall ratio of 4:5, and a rotor size of 3.0 cm), CLCMs with a particle size of 66 µm were prepared with excellent color rendering properties. Excessively large (>100 µm) and excessively small (<30 µm) CLCM particle sizes reduced the reflectance efficiency. Furthermore, the highest relative reflectivity of 32% was achieved with a 2:1 microcapsule to PVA mixing ratio. Moreover, the effect of particle size on the angular discoloration of the microcapsule coatings was investigated in detail. Coatings with particle sizes of 34–80 μm had obvious angular anisotropy, accompanied by wavelength blueshift when observation deviated from 90°. In-depth study of particle size provides a theoretical basis for the preparation of microcapsules with excellent properties. The encapsulation of microcapsules has brought liquid crystal materials closer to large-scale commercial production. However, there are few studies on the regulation of liquid crystal molecules inside microcapsules, and the need for microregion regulation is a direction for future research.

## Figures and Tables

**Table 1:** Percentage of samples and process conditions.

Samples	Percentage (wt%)		Raw Material Input (g)			Spin Speed (rpm)	Rotor Size (cm)
	717200	S5011	CLC	Gelatine	Gums		
A1	97.30	2.70	2	1.25	1.25	**500**	3.0
A2	97.30	2.70	2	1.25	1.25	**1000**	3.0
A3	97.30	2.70	2	1.25	1.25	**1500**	3.0
B1	97.30	2.70	2	1.25	1.25	1000	**2.0**
B2	97.30	2.70	2	1.25	1.25	1000	**2.5**
B3	97.30	2.70	2	1.25	1.25	1000	**3.0**
C1	97.30	2.70	2	**0.8** **0**	**0.8** **0**	1000	3.0
C2	97.30	2.70	2	**1.0** **0**	**1.0** **0**	1000	3.0
C3	97.30	2.70	2	**1.25**	**1.25**	1000	3.0
C4	97.30	2.70	2	**1.5** **0**	**1.5** **0**	1000	3.0
R	**97.75**	**2.25**	2	1.25	1.25	1000	3.0
G	**97.30**	**2.70**	2	1.25	1.25	1000	3.0
B	**96.90**	**3.10**	2	1.25	1.25	1000	3.0

**Table 2:** Process conditions of CLCM D1–D4.

Sample	Emulsifiers	Curing Agent
D1&3	PVA 1788	glutaraldehyde
D2	-	glutaraldehyde
D4	PVA 1788	formaldehyde

**Table 3:** Reflectance of CLCMs studied in the past.

Core	Shell	Fabrication Methods	Reflectivity	Particle Size	Testing Instrumentation	Ref.
RM1, RM2, NRM	Polyvinyl pyrrolidone (PVP)	Interfacial polymerization	4%	15 ± 10 μm	ultraviolet–visible spectroscopy (Lambda 750)	[11]
SLC-1717, R811	PU	Interfacial polymerization	35%	9 µm	optical fiber spectrometer (AvaSpec-2048)	[32]
E7, S5011, S811	MF	In situ polymerization	35%	10.9 µm	ultraviolet-visible spectrometer (Lambda 950)	[28]
R-CLC, G-CLC	PMMA	Solvent evaporation	25%	5–30 µm	UV/VIS/NIR spectrophotometer (UV3600)	[33]
BHR-59001, S811	Silicone methacrylate	Microfluidics	30.3%	≈160 µm	fiber-coupled spectrometer (USB 4000)	[34]
JK-1001, S811	Toluene-2,4- diisocyanate (TDI) and Tetraethylenepentamine (TEPA)	Microfluidics	45%	≈90 µm	UV-Vis-NIR spectrophotometer (Lambda 950)	[16]
CLC, chiral RM894	RM257, RM520	Reactive mesogen polymerization	23–25%	4.7 ± 2 µm	UV spectrophotometer with an integrating sphere (x-Rite color i5)	[35]
E7, R5011, S5011	RM257	Reactive mesogen polymerization	3%	60–180 µm	fiber-coupled spectrometer (USB 2000)	[29]

## Data Availability

The original contributions presented in the study are included in the article/Supplementary Materials, further inquiries can be directed to the corresponding author.

## Supplementary Materials

The following supporting information can be downloaded at https://www.mdpi.com/article/10.3390/molecules29133030/s1, Figure S1. The chemical structures of chiral compound S5011. Figure S2. (a) FTIR spectra of CLCs, CLCMs and G-A coacervates; (b) XRD pattern of CLCs and CLCMs. Table S1. Numerical comparison of average particle size, standard deviation and coefficient of variation. Table S2. Numerical comparison of average particle size, standard deviation and coefficient of variation. Figure S3. OM (top) and POM (bottom) images of CLCM RGB. Figure S4. Average thickness of microcapsule coatings of different particle sizes.
