# Supplementary material for: Reflectivity and Angular Anisotropy of Liquid Crystal Microcapsules with Different Particle Sizes by Complex Coalescence
Source: Molecules. 2024 Jun 26;29(13):3030. doi: 10.3390/molecules29133030 (PMC11242959; doi:10.3390/molecules29133030)
Supplement: Supplementary file 1 [file molecules-29-03030-s001.zip › molecules-3035593-supplementary.pdf]

Supplementary Information

Reflectivity and Angular Anisotropy of Liquid Crystal Microcapsules with Different Particle Sizes by Complex Coalescence

Yonggang Yang [1,†], Yuchen Cui [1,2,†], Yinjie Chen [1,2,*], Yanan Guo [1,2], Xiaoqi Liu [1,2], Xia Chen [3], Jianghao Liu [1], Yu Liu [1] and Zhengfeng Liu [1]

[1] School of Printing and Packaging Engineering, Beijing Institute of Graphic Communication, Beijing 102600, China

[2] Beijing Engineering Research Center of Printed Electronics Institution, Beijing Institute of Graphic Communication, Beijing 102600, China

[3] School of New Media, Beijing Institute of Graphic Communication, Beijing 102600, China

* Correspondence: chenyinjie@bigc.edu.cn

† These authors contributed equally to this work.

1. Chiral dopant S5011

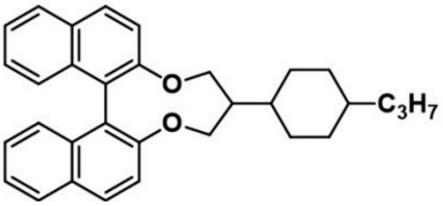

Figure S1. The chemical structures of chiral compound S5011.

2. FTIR and XRD characterization

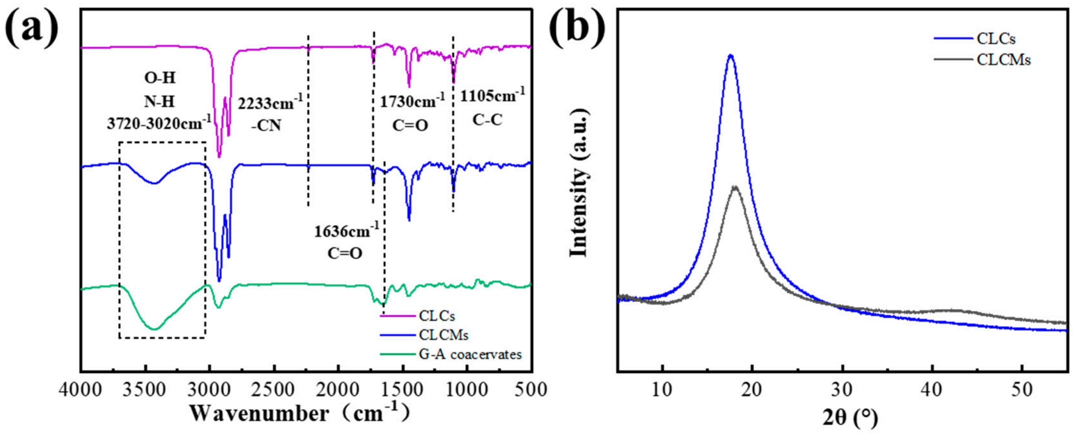

Figure S2. (a) FTIR spectra of CLCs、CLCMs and G-A coacervates; (b) XRD pattern of CLCs and CLCMs.

From Figure S2a, the absorption bands of G-A aggregates at 3720-3020 cm^{-1} are caused by the stretching vibrations of O-H and N-H in G-A aggregates. The absorption peak of 3000-2750 cm^{-1} is caused by the C-H stretching vibration on the saturated alkane $-CH_3$ and $-CH_2$ in G-A aggregates. The absorption peak at 1636 cm^{-1} corresponds to the stretching vibration peak of C=O in the amide group. The absorption peaks of CLCs at 1105 cm^{-1} were caused by in-plane vibration of C-H in cholesterol derivative molecules and the vibration of C-C single bond skeleton, and the absorption peaks at 2233 cm^{-1} were characteristic of the cyano group. The characteristic peak of CLCs at 1730 cm^{-1} is the stretching vibration of C=O in the cholesterol derivative molecule. The absorption peaks of CLCMs appear at 3720-3020 cm^{-1}, 2233 cm^{-1}, 1730 cm^{-1}, 1636 cm^{-1}, 1105 cm^{-1} corresponding to the characteristic peaks of CLCs and G-A aggregates. The above proves that the microcapsules are prepared successfully.

XRD tests were performed in Figure S2b. The CLCs exhibit intensive diffraction peaks located around 20°, which is attributed to the orientational order of layered arrangement. The CLCMs had the same strong diffraction peak at 20° as the CLCs, proving that the synthesized liquid crystal microcapsules still maintain the chiral layered arrangement [1]. Based on the first order Bragg diffraction as following,

$$2d \sin\theta = n\lambda \tag{S1}$$

where n is equal to 1.5, λ is the wavelength of incident X ray 0.154 nm. The interlayer spacing d of CLCs can be figured out as 3.38 Å.

3. Standard deviation and coefficient of variation

The standard deviation (σ) and coefficient of variation (C_v) are calculated as follows,

$$\sigma = \left[\sum_{i=1}^{n} \frac{(d_i - \bar{d})^2}{n}\right]^{\frac{1}{2}} \tag{S2}$$

$$C_v = \frac{\sigma}{\bar{d}} * 100\% \tag{S3}$$

where d_i, $\bar{d}$ and n are diameter of single CLCMs, average diameter of CLCMs and number of statistic CLCMs.

Table S1. Numerical comparison of average particle size, standard deviation and coefficient of variation.

Sample	Average particle size (μm)	Standard deviation (μm)	Coefficient of variation (%)
A1	123.43	58.20	47.2
A2	66.45	21.84	32.9
A3	34.06	17.22	50.6
B1	191.19	62.61	32.8
B2	111.86	44.97	40.2
B3	66.45	21.84	32.9
C1	48.44	15.72	32.4
C2	64.33	19.98	31.1
C3	66.45	21.84	32.9
C4	67.86	24.71	36.4

Table S2. Numerical comparison of average particle size, standard deviation and coefficient of variation.

Sample	Average particle size (μm)	Standard deviation (μm)	Coefficient of variation (%)
D1&3	68.54	22.98	33.5
D2	90.18	51.86	57.5
D4	62.14	22.62	36.4

4. OM and POM images of CLCM RGB

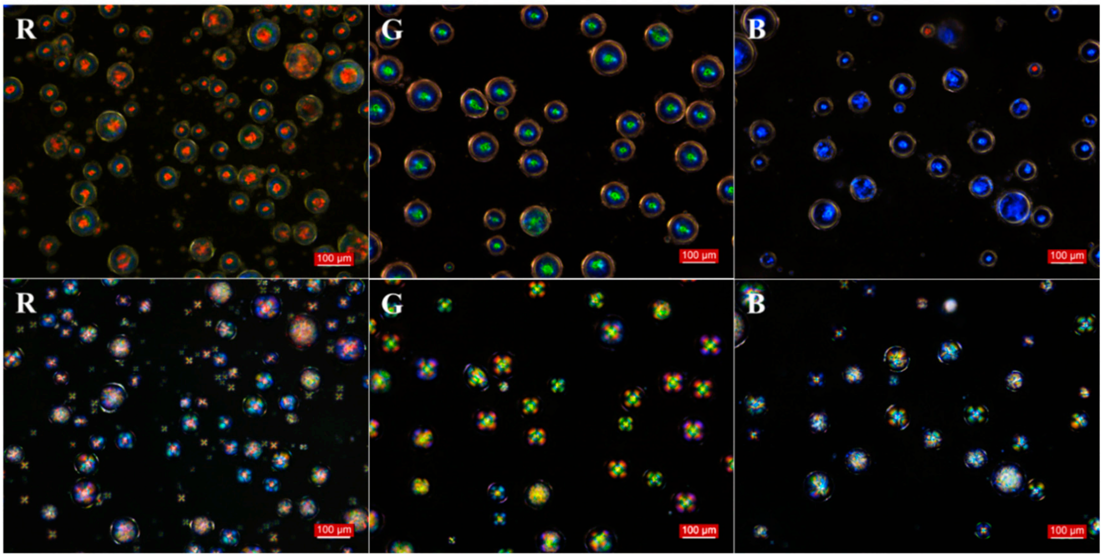

Figure S3. OM (top) and POM (bottom) images of CLCM RGB.

5. Average thickness of microcapsule coatings of different particle sizes

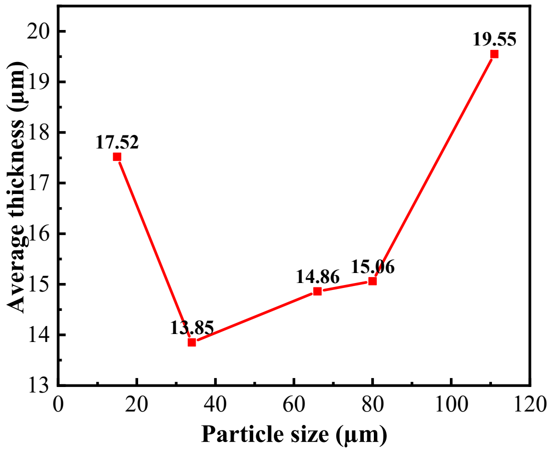

Figure S4. Average thickness of microcapsule coatings of different particle sizes.

Reference

1. Lin, P.; Yan, Q.; Wei, Z.; Chen, Y.; Chen, S.; Wang, H.; Huang, Z.; Wang, X.; Cheng, Z. Chiral photonic crystalline microcapsules with strict monodispersity, ultrahigh thermal stability, and reversible response. *ACS Appl. Mater. Interfaces* **2018,** *10,* 18289-18299.
